# Critical analysis of evidence about the impacts on surgical teams of ‘mental practice’ in systematic reviews: a systematic rapid evidence assessment (SREA)

**DOI:** 10.1186/s12909-020-02131-3

**Published:** 2020-07-14

**Authors:** Huon Snelgrove, Ben Gabbott

**Affiliations:** 1 Department of Education and Development - GAPS Simulation & Skills Centre, St George’s University Hospitals NHS Foundation Trust, London, UK; 2 Ben Gabbott Trauma and Orthopaedic Department, St George’s University Hospitals NHS Foundation Trust, London, UK

**Keywords:** Mental practice (MP), Mental imagery (MI), Mental rehearsal (MR), Surgical training, Surgical teams, Medical education, Learning, Team training

## Abstract

**Background:**

Mental Rehearsal (MR) the cognitive act of simulating a task in our heads to pre-experience events imaginatively. It has been used widely to improve individual and collective performance in fields outside healthcare and offers potential for more efficient training in time pressured surgical and medical team contexts. The study aims to review the current systematic review literature to determine the impact of MP on surgical performance and learning.

**Methods:**

Medline, Embase, British Educational Index, CINAHL, Web of Science PsycINFO, Cochrane databased were searched in the period 1994–2018. The primary outcomes measure were performance improvements in surgical technical skills, stress reduction, confidence and team performance. Study quality of the Systematic Reviews was assessed using AMSTAR 2, a critical appraisal tool for systematic reviews. The reported impacts of MP in all included studies were mapped onto Kirkpatrick’s framework for the evaluation of educational interventions.

**Results:**

Six Systematic reviews were identified which met the inclusion criteria, of which all reported positive and varying benefits of MP on surgical performance, confidence, and coping strategies. However, reported impacts on a modified Kirkpatrick’s framework did not exceed level 3. Mental practice was described in terms of mental imagery and mental rehearsal with most authors using each of the terms in their search strategies. The impacts on transfer to practice and the long- term acquisition of skills, but also personal uptake of mental practice routines were not reported.

**Conclusion:**

The majority of studies demonstrate benefits of MP for technical performance. Overall the systematic reviews were of medium to high quality. However, studies lacked a sufficiently articulated evaluation methodology to examine impacts beyond the immediate experimentations. This is also due to the limitations found in the primary studies. Future research should look at longitudinal mixed method evaluation designs and focus on real clinical teams.

## Background

Practising surgical skills is one of the most crucial tasks for both the novice surgeon learning new procedures and surgeons already in practice learning new techniques or preserving acquired ones. The increased complexity of surgical procedures has heightened interest regarding how to help surgeons attain, enhance and maintain expert performance more effectively.

In recent years the question of what constitutes effective surgical education for all levels of experience has driven increasing interest in the use of various modalities of clinical simulation. However, technology-based simulations with VR and or mannequins are expensive, resource intensive and time consuming. Mental practice (MP) is purported to be an alternative simulation approach to enhance performance.

Mental practice (MP) is most commonly described as the ‘symbolic’ mental rehearsal of a task in the absence of actual physical rehearsal [1–3]. Fittingly, it is closely aligned with a notion of consciousness entailing the setting up and planning of future goals [4]. Evidence in a variety of fields spanning elite sports and music to neuro-rehabilitation have lent substance to the idea that MP can be effective in improving practice [5–10]. Interestingly, the research also suggests that the process of MP may be either individual or coordinated with others [8, 11–15].

**Fig. 1:**
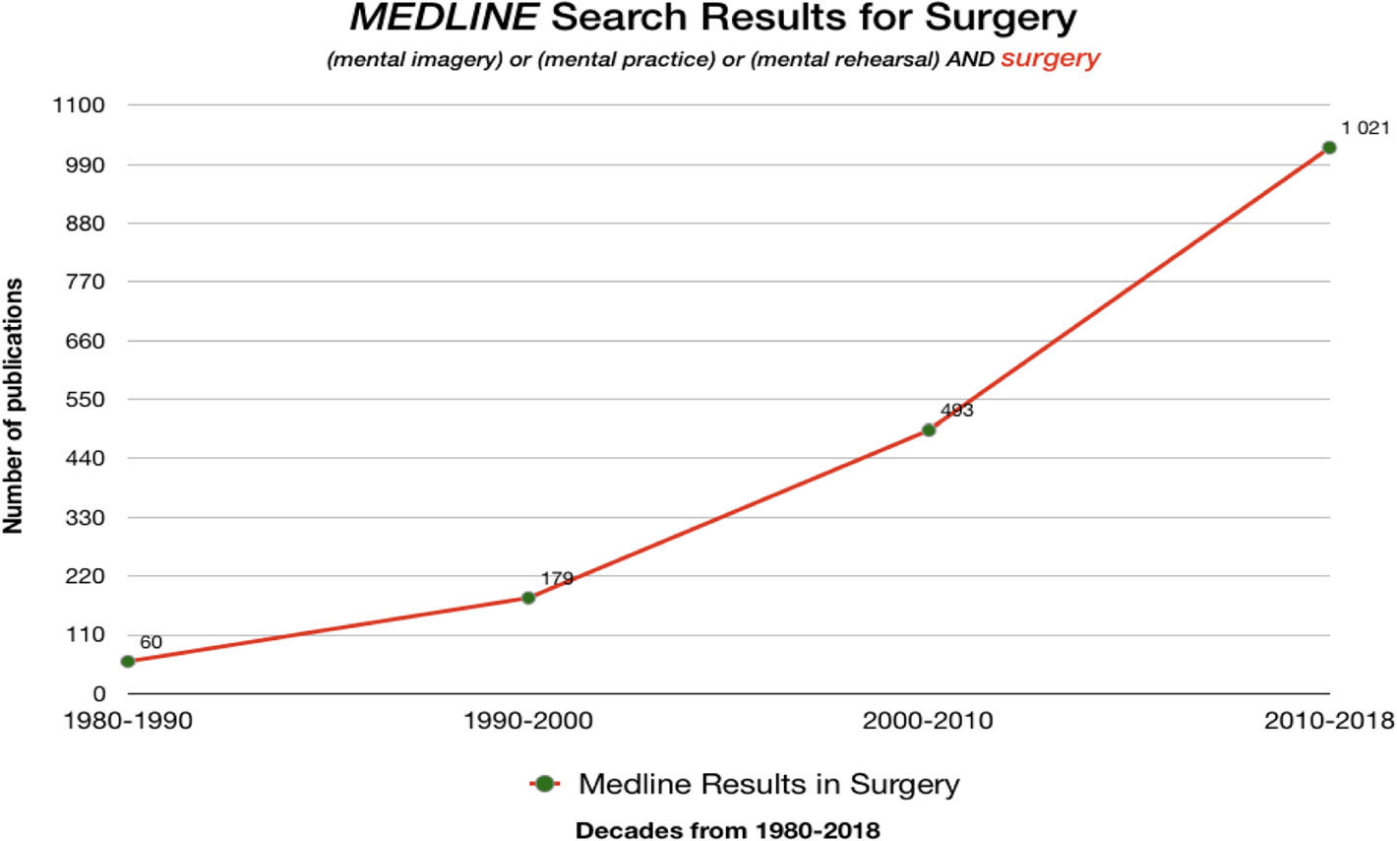
Medline Results for Surgery – decades from 1980 to 2018

The concept of mental practice (MP) subsumes notions of rehearsal, mental imagery and mental simulation and the ever-increasing number of publications in surgery attests to surgical educators’ enduring expectations that it is beneficial (Fig. 1).

Proponents of MP claim it offers a number of potential benefits for surgeons at all levels of training [16]. It may for example augment physical practice in improving accuracy and precision of surgical movements [17]. An early meta-analysis by Driskell et al. [3] gave an explanatory mechanism for this suggesting that by imagining how a movement looks, feels and affects a patient MP may strengthen cortical representations of the task performed by previous physical practice, or may prime specific neuromuscular pathways.

More recent notions of mental practice in the literature go beyond an exclusive focus on individual psychomotor skills and they embrace a broad range of cognitive skills too. These range for example from mental readiness, risk assessment, and anticipatory planning to a wide spectrum of other non-technical skills such as team work, coping strategies, situational awareness and task management [8, 18–24]. These skills are therefore individual but also emerge from social interactions with other team members. Nevertheless, there is still some confusion about the boundaries of what constitutes simulation through mental practice, and the degree of interplay between these broader social cognitive skills and a narrower focus on psychomotor skills [25–28]. Agreement exists however that each is important for effective surgical practice as each captures cognitive skills that resonate on a very practical level of surgical work.

In this article we construe mental simulation as encompassing mental rehearsal activities undertaken by practicing surgeons prior to performing a surgical task to improve their knowledge, skills and behaviour and with the aim of enhancing overall surgical performance and how it is planned and organized.

Mental practice activities vary in format, content, duration and setting. As a result, surgical educators have to make choices when designing and delivering training to improve performance, either at individual or on a team level. This includes interventions developed for training purposes in simulated contexts but also priming exercises prior to actual surgical procedures. In these circumstances research evidence about the impacts of ‘mental practice’ has a useful role in shaping decisions about what kind of mental practice may be effective for which groups of practitioners in which contexts. Systematic reviews of research about impacts can provide a comparatively efficient and rigorous source of evidence for this purpose and ensure that future research on education meets the criteria of scientific validity, high-quality, and practical relevance that is sometimes lacking in existing evidence on educational activities, processes, and outcomes [29].

This study presents the findings and discusses the implication for practice and research of our analysis of a number of systematic reviews of ‘mental practice’. These reviews were identified to answer the following questions: which kinds of mental practice lead to what outcomes for what kinds of surgical practitioner?

## Methods

We identified systematic reviews of ‘mental practice’ in surgery which, together with the studies included in them provide a comprehensive view of the research literature on the impact of mental practice on surgical teams.

We used an approach that has been termed a systematic-rapid evidence assessment (SREA) [30]. This approach follows the principles of systematic review but a number of strategies are used to accelerate a more rapid review process. Specific adaptations used in this SREA included a selective data extraction process, limited quality assessment process, and simple synthesis of included materials. In addition, we undertook a ‘review of reviews’ method, as opposed to reviewing primary research studies. A SREA approach retains the advantages of transparency and rigor in the review process compared with non-systematic literature reviews; however, it reduces the time resources required when undertaking a comprehensive systematic review of primary research. The specific procedures used in this SREA are detailed below.

### Locating systematic reviews

We searched a range of sources including bibliographic databases (BEI, ERIC, Medline, CINAHL PsycINFO, Web of Science); the internet (Google Scholar); and systematic review organisations, specifically the Cochrane EPOC group, The Best Evidence in Medical Education (BEME) network, and the Joanna Briggs Institute. A search string comprising a variety of synonyms for ‘mental practice’ – ‘mental imagery’, ‘mental rehearsal’, ‘mental simulation’ – was combined using Boolean term ‘or’, the results of which were then combined with ‘surgical teams’ and ‘systematic review’. We conducted the literature search between July 2018 to December 2018.

### Selection criteria

**Fig. 2:**
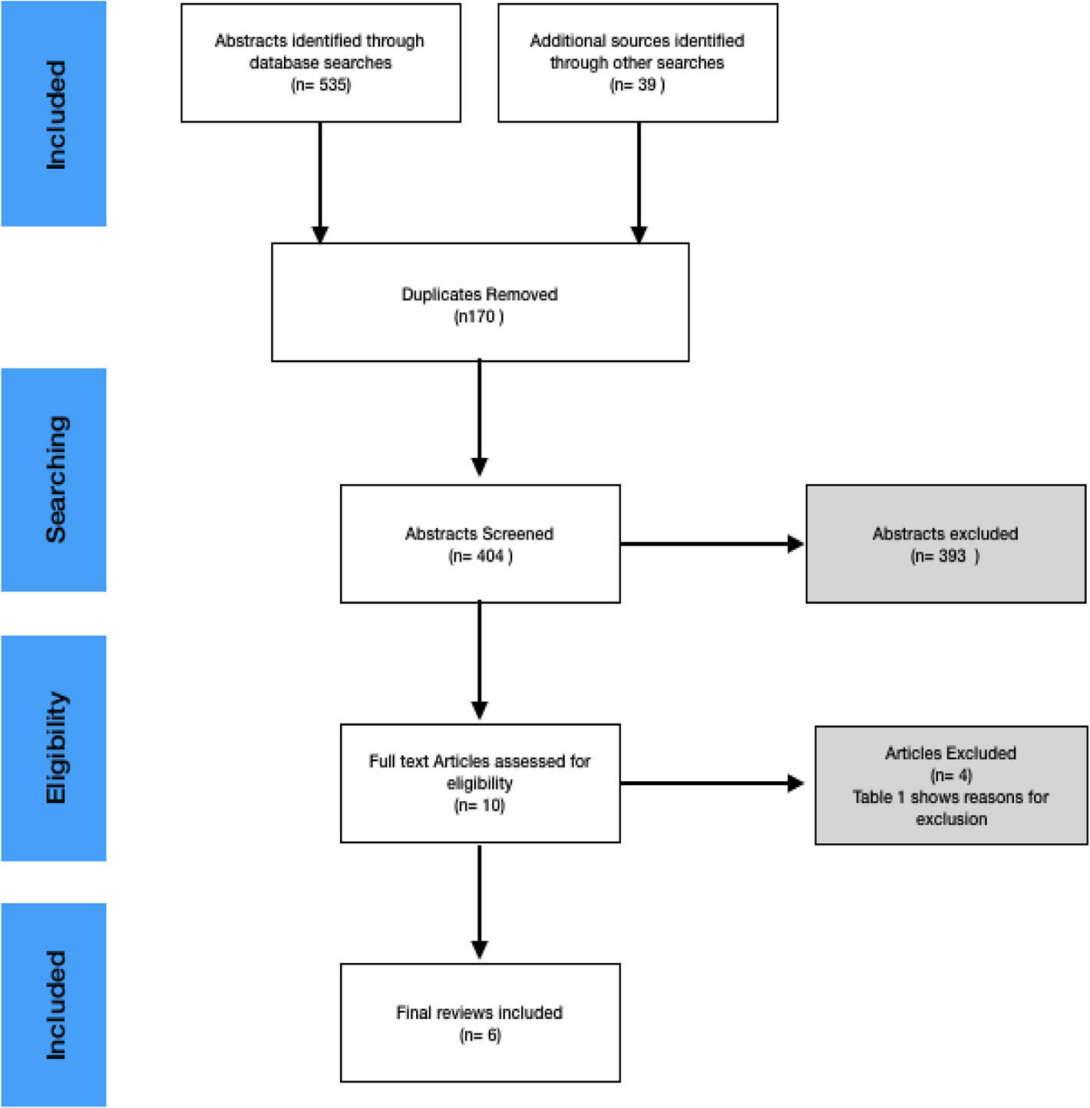
Prisma Flow Chart

The following selection criteria were applied to titles and abstracts of provisionally identified papers to identify relevant reviews [1]: reviews investigating the impact of mental practice on surgeons or members of surgical teams [2] systematic reviews (i.e. reviews of research with recognised review methods reported [3] reviews in English, French or Italian since 2000. A review had to meet all these criteria to be included. At the second stage of screening (full papers) these selection criteria were reapplied. Reference lists of included studies were scrutinized for additional papers. Figure 2 provides an overview using the PRISMA framework [31]. One author conducted the initial search for literature and the initial exclusion stage (HS). Both authors then reviewed the remaining titles, abstracts, and full texts for eligibility and extracted the relevant information (HS, BG).

### Data extraction, analysis, and synthesis

Reviews that met the selection criteria were coded for relevant details about contexts, methods, and review results or outcomes and a narrative synthesis of each study was written. This narrative involved summarising and combining the descriptive and contextual outcome information from the included reviews. The reported outcomes were categorised using a version of Kirkpatrick’s Evaluation model adapted by Simpson and Scheer for surgery [32] to gauge the overall quality of the evidence (Table 1).

**Table 1:** Kirkpatrick’s Framework

Key Outcomes	Kirkpatrick Evaluation Levels
**Outcomes**	**Descriptor**
1. Reaction	Participant’s views on the learning experience, its organization, presentation
2a. Learning/change in attitudes	Changes in attitudes or perceptions among participant groups towards teaching and learning
2b. Learning/Modification of Knowledge or skills	For knowledge, this relates to acquisition of concepts, procedures and principles; for skills, this relates to the acquisition of thinking/problem solving, psychomotor, and social skills
3. Learning/Behavioral Change	The transfer of learning to the workplace (i.e. surgical practice) or willingness of learners to apply new knowledge and skills.
4.a Change in the system/organizational practice	Wider changes in the organization, attributable to the practice of MP
4b Changes among learners	Changes in healthcare learning performance as a result of training activities
4c Benefits to patients/ communities	Benefits to patients/wider public/communities as a result of faculty development

The Kirkpatrick model defines four levels of training evaluation. As Table 2 shows, levels 1, 2a, 2b, and 3 cover outcomes that measure the impact on the surgical team members who participated in a ‘mental practice’ intervention, whereas levels 4a, 4b, and 4c measure impacts on organisational practices (e.g. dedicated times, pre-surgical briefings, guidelines and training documents, collective perceptions of safer practice, sustained learning and or the quality of patient care).

**Table 2:** Excluded reviews

Study	Reason for Exclusion
Pike et al. (2017) [24]	Focussed on physical simulation-based tasks as mental practice in pre-operative intervention to ‘warm up’
Marcus et al. [33]	Non-systematic narrative review
Shearer [34]	Non-medical focus
Hall (2003) [5]	Non-systematic narrative review

### Quality assurance processes

The search strategy and inclusion criteria were tested and developed iteratively by the two authors. Screening of abstracts was done by one of the authors with a random 10% sample screened by a second author. Any discrepancies were discussed until consensus was reached. Data extraction and coding of the key information was undertaken by one of the authors (HS). A second author checked the extracted information for consistency and conducted a short quality assessment review of 3 of the selected systematic review using AMSTAR-2 [35] which stands for ‘A MeaSurement Tool to Assess systematic Reviews’. Once again, discrepancies were discussed and resolved for each study.

## Results

Our literature searches initially generated 535 abstracts (Fig. 2). A detailed key word search result table is produced in Supplementary materials. After removing duplicates, the titles and abstracts of 404 studies were screened applying the criteria described above. Ten full text articles were assessed for eligibility, of which four were subsequently excluded (Table 2). Six systematic reviews of ‘mental practice’ were included in the analysis [24, 36–41].

### Stated aims and conceptual field

The aims of the studies and the terms authors used to encompass ‘mental practice’ are shown in Table 3. The stated aims of the reviews ranged from searches to identify effective designs of mental practice tasks, to the application of mental practice to improve surgical education, to comparison of impacts of MP on performance of novices and experts, to improvements in technical confidence as well as stress reduction, and lastly to prime surgical performance in ‘warm up’ routines.

**Table 3:** Terms encompassing ‘mental simulations’ in surgery or surgical education in review

Studies	Stated Review Aims	Key Words	N studies
Schuster et al. [40]	To identify the characteristics of ‘mental imagery training sessions’ (MITS) ‘with *positive* results’ and compare across disciplines and identify ‘fundamental intervention designs’	*Mental imagery, mental practice, mental rehearsal, mental movements, eidetic imagery, visual imagery, guided imagery; motor imagery, mental training.*	133
Cocks et al. [38]	To explore how the specific principles of mental practice can be applied to the improvement of surgical performance – both in novice and expert surgeons.	*Mental practice, mental imagery, mental rehearsal, motor rehearsal.*	10
Sevdalis et al. [41]	To explore the role of mental imagery and mental practice in surgical training and performance.	Mental practice, mental imagery, mental rehearsal.	13
Rao et al. [37]	To evaluate the role of mental training in the acquisition of surgical technical skills.	*Mental training, mental imagery, technical skills, surgical training.*	9
Davison et al. [39]	To determine the role of mental training in surgical education and the feasibility of its incorporation in surgical curricula.	*Cognitive training, mental training, mental rehearsal, brain training.*	14
Anton et al. [36]	To identify how mental skills training has been applied in surgery and examine its effectiveness in enhancing surgical performance and reducing stress.	*Mental practice, mental rehearsal, mental imagery, mental readiness, mental competency, Mental skills training; performance enhancement; stress management training; stress coping*	19

The key words used by authors to describe the conceptual domain are for the most part linguistically unmarked, the most common imbrications being ‘mental practice’, ‘mental imagery’ and ‘mental rehearsal’. The less common and more marked concepts included terms such as ‘mental simulation’, ‘eidetic imagery’, motor imagery’ and ‘visual imagery’.

### Mental practice content and process

An outline of mental practice activities contained in the included reviews is presented in Table 4. All six reviews attempted to assess surgical performance in relation to MP and its impact on psychomotor execution of the surgical task. Table 4 lists the variety of validated rating scales the reviews identified for both technical and non-technical skill ratings.

**Table 4:** Information on Mental Practice activities

Review Citation	MP Focus	Evidence of Change Tools	Learning Activities	Duration/Range
Shuster et al.* 2011 [40]	Cognitive and motor task related activities for surgeons	Pre post tests	Laparoscopic cholecystectomy; Surgical cystoscopy	30–50 min
Cocks et al. 2014 [38]	Cognitive and motor task related activities for surgeons. Stress management	GRS GSOP OSCE OSATS OTAS	Basic suturing Surgical cystoscopy Cricothyrotomy Laparoscopic cholecystectomy* Carotid endarterectomy*	8–30 min
Sevdalis, et al. 2013 [41]	Cognitive and motor task technical skills for surgical trainees and non-technical skills (stress coping)	Pre post tests OSATS GSOP Observation tools Simulator-derived parameters ISAT OTAS	Basic suturing Surgical cystoscopy Cricothyrotomy Laparoscopic cholecystectomy* Carotid endarterectomy*	5–30 min
Rhao et al. 2015 [37]	Cognitive and motor task technical skills for surgical trainees.	Time/trajectory measurements Visual Imagery Test (RMPFBT) OTAS MIQ Imperial Stress test OSCE	Cutting circle in box trainer Incision and suture of live anaesthetised rabbit Pelvic box simulator Simulated LC. VR LC Simulated Lap’ knot-tying. Cricothyroidotomy on mannequin	3–90 min
Anton et al. (2017) [36]	Cognitive skills training to enhance surgical technical and non-technical performance in the OR or simulator	Surgeon procedure specific GRS rated Time precision accuracy FLS HRV MHPTS OSATS OTAS STAI MIT Qualitative analyses	FLS box trainer VRS surgical tasks	No analysis of time in review
Davison et al. 2017 [39]	Cognitive skills training to enhance surgical technical and non-technical perform-ance in the operating room or simulator	Global rating scales Peg transfer times Assessor scores MIQ	Peg transfer on L simulator VRS surgical tasks Box trainer MI relaxation	3–90 min

* Synthesis of selected studies in surgery included in multi-field review examining MP In ‘Education’

In general, the reviews defined ‘mental practice’ with variable emphases. Three studies focussed on cognitive and motor task activities, but also considered the effects of MP on managing non-technical skills, and in particular the management of stress [36, 38, 41]. Differences across studies emerge concerning how broadly or circumscribed the nature of MP is defined. One review investigating optimal educational designs to improve motor coordination ‘of moving body parts’ described MP as an umbrella term covering ‘mental imagery’ and broader mental ‘training’ interventions [40]. While most of the reviews assume in varying degrees that MP is a given capability among subjects, two argue that MP may depend on imagery strength and the neural networks underlying imagery [40, 41]. For instance, Sevdalis et al. [41] argue that individual differences across the population in visual representation strength determines how MP impacts performance. According to these authors, groups therefore need to be separated on the strength of their imagery using validated tools such as the Mental Imagery Questionnaire [5, 42].

Although the reviews reported MP practices in medicine, two reviews compared MP across different domains. Cocks et al. [38] compared MP in medicine and sports psychology to see what insights could be applied to both novice and experienced surgeons. Schuster et al. [40] examined MP across five different domains – education, medicine, music, psychology and sports. The authors included only two studies with practising surgeons and another seven with undergraduates or nurses which they organised under ‘educational studies’. A further 37 studies on MP in rehabilitation with patients were included under ‘Medicine’. MI activities were described as cognitively-focussed or task-oriented and all the included studies measured change with a pre-post test. Activities using MP spanned sterile glove removal to laparoscopic cholecystectomy. MP was used before physical practice with a duration across trials of less than 30 min. The mode of MP was not reported.

According to the included reviews, most interventions were carried out in simulation laboratories, with very few in clinical contexts. The use of control groups and comparison interventions is consistent across the studies reviewed as are common standard outcome measures in surgery. Table 4 lists the different measures. The most commonly used evidence of change tools following MP were the Objective Structured Assessment of Technical Skills and the Observational Teamwork Assessment for Surgery rating scales [42, 43]. Tools used in adjunct to these comprised observational Global Rating Scales for operative performance measures, [36, 38, 39, 41] measures of stress [36, 37, 40, 41] and lastly mental imagery measures [36, 37, 39, 41].

In contrast, less comparability is possible in descriptions of the delivery mode of MP. These included MP delivered in groups, delivered one-to-one, with the adjunct of video and written scripts, MP directed and taught formally (face to face) and undirected or self-directed MP by participants at home. MP was also embedded in an assortment of other activities which ranged from relaxation with hypnosis, talk aloud protocols and finally with teaching underpropped by psychologists or MP experts. One review’s account of MP was wrapped in a miscellany of warm-up activities consisting of techniques for attention management, stress control, and goal-setting [36]. As a result, it was difficult to discern within and across the reviews the exact nature of the impacts of MP. Or rather it was difficult to see the boundaries of MP with other kinds of stimuli to shape performance. Populations too were heterogeneous ranging from first year undergraduates to novice trainees to senior surgeons.

More importantly, the reviewers often provide little descriptive detail of how MP was enacted as an intervention. It is not clear whether the lack of description reflected a lack of detail in the primary studies or in the systematic review process. Others have already reported a lack of detail to be a common feature of MP studies, along with unsatisfactory experimental designs [41, 44].

### Reported impact on learning outcomes

Of 73 experimental reports contained in the reviews, fifty-one reported positive impacts of MP while 16 described no effect, or negative effects on performance. We classified the reported outcomes in each systematic review using a modified outcomes typology [32] as shown in Table 5. The table lists the number of reported impacts from primary studies synthesised in the reviews.

**Table 5:** Kirkpatrick Evaluation Framework

Study	Level 1	Level 2a	Level 2b	Level 3	Level 4a	Level 4b	Level 4c
Schuster et al. [40]	–	2	7	1	–	–	–
**Cocks et al.** [38]	1	4	6	2		–	–
**Sevdalis et al.** [41, 44]	3	3	7	1	–	–	–
**Rao et al.** [37]	2	2	4	–	–	–	–
**Davison et al.** [39]	6	3	3				
**Anton et al.** [36]	5	7	11	4	–	–	–

Looking at Level 1, there is little description in the reviews of how reactions were collected. Reactions cover the learners’ views of the learning experience, its organisation, presentation, content, teaching methods and quality of instruction. Where personal reactions were captured in primary studies these were related to use of the MIQ tool.

Changes in learner confidence and improved stress reduction strategies are assimilated into Level 2 with the implication that MP changes participants’ conceptions of how to prepare, mentally rehearse or simulate tasks.

What emerges from this synthesis is that the majority of outcomes are at level 2a (Learner perceptions) and 2b (Knowledge & Skills acquisition) where the reported evidence base is stronger and intervention groups significantly outperformed controls. Measurements were made using a combination of validated instruments such as the Observed Structured Assessment of Technical Skills (OSATS), Global Rating Scales, and stress related measures.

**Fig. 3:**
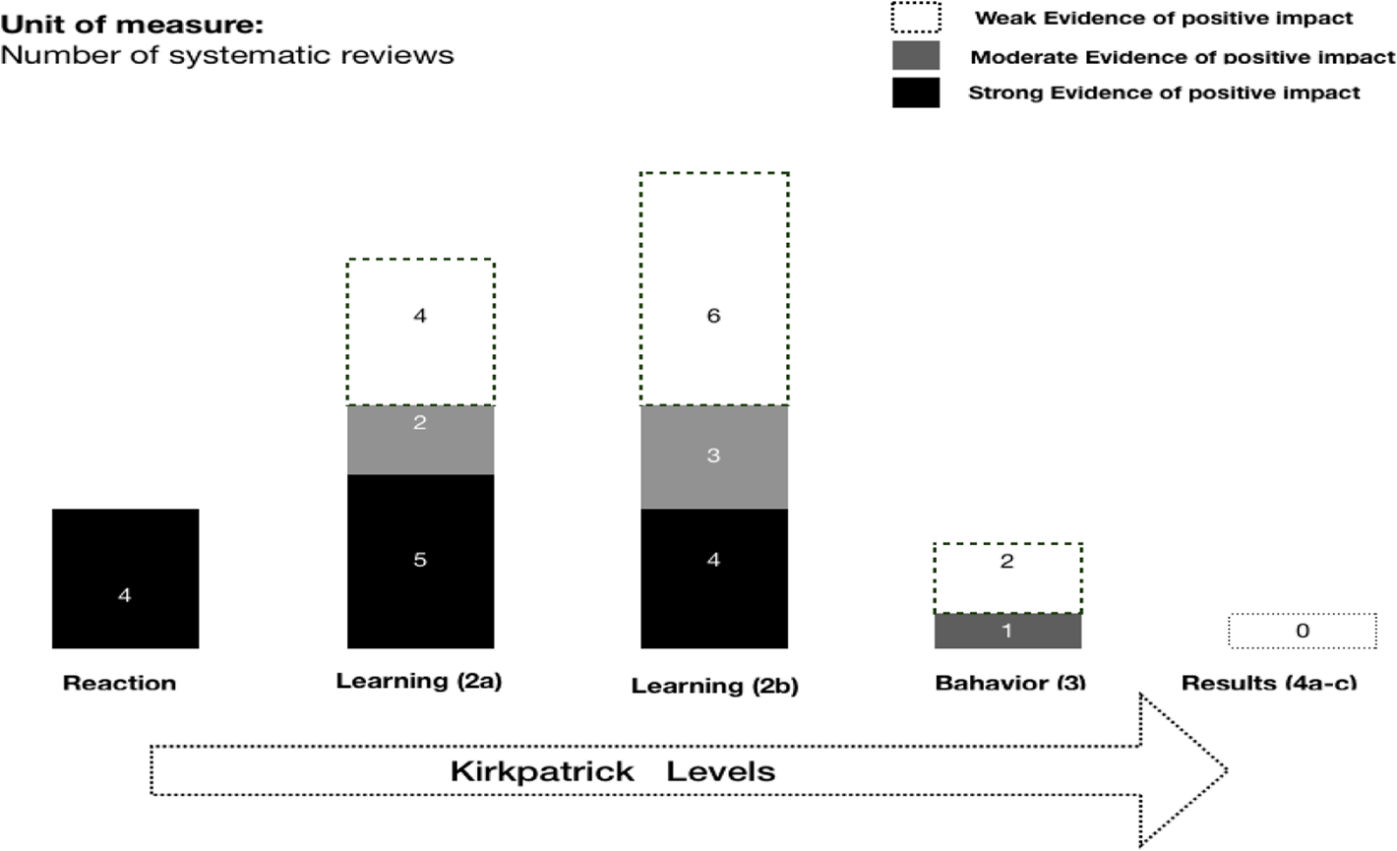
Overview of the application of the Kirkpatrick Model

Figure 3 shows an overview of the application of the Kirkpatrick model to the selected studies. The number of reviews classifying the quality of data at each level is enumerated in each column. This shows that evidence for positive impacts of MP is strongest at the Level 2a and 2b, which relates to perceptions by learners of positive impacts (confidence, emotions) and psychomotor performance immediately following the MP intervention. (See also Table 2).

Only three reviews referred to evidence for behaviour change (Level 3) for longer-term impacts of MP – e.g. sustained acquisition of psychomotor skills, knowledge and coping strategies - although in all three, reviewers expressed concerns for data quality. None of the studies reported results at Level 4 (Changes in practice at system level). However, some sporadic descriptions of positive trends in transfer of learning emerge across the reviews. Cocks et al. [38] report one study of learning ‘transfer’ where MP and physical practice in combination, improved medical students suturing skills at distance of time on anaesthetised rabbits compared to controls who did physical practice alone. Similarly, Anton et al. report a recent study in endovascular surgery [45] in which error rates during the endovascular phase were lower after the MP intervention compared to before. Other reviewers [37, 40] cited evidence of positive long-term impacts and transfer at Levels 3–4 in populations excluded from their selection criteria: for example, patients in neuro-rehabilitation and pain management [9, 46]. Tellingly, all of these studies applied longitudinal and mixed method designs for evaluation of impacts with significant improvements compared to control groups.

Generally, reviewers struggled to find details of follow up reports on the role of MP as a process and technique for fine honing individual or collective learning. Perhaps less justifiably there was also little detailed discussion of methods of how this may be achieved in future research. Nevertheless, three reviews did advocate the need for prospective investigations into impacts which included outcome measures derived from systematic ethnographic observations, follow-up self-reports and evidence of changes in local training methods, team routines or curriculum [37, 38, 41].

### Quality issues in the systematic review

An outline of the methods used in the reviews is presented in Table 6. All six reviews reported using standard systematic review methods for searching, selecting, analysing and report writing.

**Table 6:** Review Methods and AMSTAR 2 Rating

Citation	Study Selection Criteria	Searches and Results	Synthesis Method	AMSTAR 2
Shuster et al. (2001) [40]	Any design on Motor skill and mental imagery in Music, Medicine Education Psychology, Sports.	Search dates 1966–2007. 24 data bases searched. (CINAHL Cochrane, PsycINFO, ERIC, BEI, SCOPUS) 795 abstracts screened for medical education. 9 included studies	Meta-analysis not possible. Trend analysis to identify MI interventions with positive results	High Quality
Cocks et al. (2014) [38]	MP in surgery (RCTS only)	Searched, PubMed, Medline, PsycINFO, Embase. 83 abstracts screened in sport and surgery and 10 studies on surgery only included	Not reported	Moderate
Sevdalis et al. (2013) [41]	Cognitive and motor task technical skills for surgical trainees	Searched Medline and PsycINFO databases to 2012 13 studies included	Not reported	High Quality
Rhao et al. (2015) [37]	MP in surgery (RCTS only) for surgical technical skills	Searches dates: up to 2014.The 7 databases searched: Medline; Embase; Web of Science; Clinical trails.gov.org; SIGN guidelines; NICE guidelines; Cochrane; and cross references of retrieved studies. 186 studies were included for title and abstract review, 18 full texts reviewed and 9 included.	Meta analysis not possible. Jadad scoring for RCT quality used.	High Quality
Anton et al. (2017) [36]	Mental skills training in surgery for technical and non-technical skills	Search dates from 1996 to 2016 on 3 databases: Medline, PsycINFO, Clinical Key.	Not reported	Low
Davison et al. [39]		Two databases searched, Medline and Embase. Period not specified.	Not reported	Moderate

*AMSTAR 2 - Systematic Review Rating Scale: *High Quality. Moderate Quality Low Quality Critically Low Quality*

All the reviews undertook searches on several bibliographic databases. Two studies included searches of reference lists of included articles, journal hand searches, and expert recommendations. One study searched for studies that were not published in academic journals by searching the internet.

Of the 73 studies that we identified in the reviews as being an included study, only 13 appeared in more than two of the reviews. This seems quite low given many of the studies were published after 2010.

A closer look at the selection criteria used in the reviews suggests this was possibly due to specific selection criteria, for example, the inclusion or not of specific activities under the term ‘mental practice’. Nevertheless, the development of findings of the earlier reviews was generally reiterated in the reporting of subsequent reviews.

All of the reviews reported some form of quality assessment, but there was considerable variation. For example, only two reviews offered explanations of how the scoring was operationalised to measure possible threats to validity (bias) or how the scores awarded to included studies were calculated [37, 40]. The remaining four studies reported no synthesis method.

The use of systematic approach to synthesis is an important element in any systematic review. In the only two reviews where the synthesis method was described as meta-analysis, [37, 40] this synthesis proved impossible because of the heterogeneity of the primary studies. The authors subsequently used trend analysis and Jadad [47] scoring of RCTs to rate the quality of the studies. It was not clear in any of the other reviews how study quality assessment ratings were incorporated in the process of synthesising results from individual studies (e.g. through weighting or individual reviewer’s interpretation).

To further support our critical appraisal of secondary studies we used the AMSTAR 2 critical appraisal tool for systematic reviews [35]. AMSTAR is not intended to generate an overall score but can be used to indicate four broad levels of quality: Critically Low, Low, Moderate and High Quality. It has 16 items with an overall rating based on weaknesses in critical domains. Two authors independently applied the tool to the reviews and came to a consensus through discussion whenever discrepancies emerged. For example, these occurred in 2 papers [36, 39] when collating factors to determine a ‘moderate’ or ‘low rating’ using the AMSTAR tool.

Table 6 shows that three of the reviews were rated as ‘High Quality’ using the tool, two of ‘Moderate Quality’ and one of ‘Low quality’. None of the studies satisfied criteria for a rating of Critically Low Quality’.

### Theoretical perspectives

All six reviews referred explicitly to theory to provide explanatory frameworks for how MP enhances learning and performance. Table 7 provides an overview.

**Table 7:** Reference to Theory

*Review Citation*	*Theory of Change*
Schuster et al. (2011) [40]	Functional Neuroscience & Holmes and Collins (2001) PETTLEP model
Cocks et al. (2014) [38]	Cognitive imagery; self-efficacy; positive thinking (mental readiness); deliberate practice and feedback.
Sevdalis et al. (2013) [41]	Neuromuscular model; cognitive psychology - Bio informational Theory.
Rao et al. (2015) [37]	Dual code theory; neuroplasticity.
Davison et al. (2017) [39]	Neurophysiology; neuroplasticity; self-efficacy; expertise acquisition.
Anton et al. (2017) [36]	Cognitive load theory and stress; goal setting and motivation theory

Theories were drawn from a broad field stretching from Neurophysiology to motivational psychology to goal-setting and to educational and feedback theories of learning. Theories of Psychological cognition (e.g. Dual Coding Theory) and the bases of MP in Neurophysiology were referred to most commonly; the later draws on empirical evidence of neuroplasticity and the proposition that learning is reinforced when the brain activates neuronal pathways to simulate or ‘rehearse’ physical actions. While reviews made reference to their own theoretical presuppositions, there was little synthesis of theories espoused in the primary research they had examined. It is not clear therefore to what extent primary studies were moored or unmoored to any discernible theoretical underpinning.

## Discussion

The use of the Kirkpatrick Model allowed us to identify what type of evidence was available for MP, and to what extent reviews demonstrated the effectiveness of training intervention. In our analysis, we used the model as an educational heuristic and did not assume causal links between the levels.

Consistent with previous evidence on the potential value of mental practice in sports psychology and neurobiology, two-thirds of primary studies reviewed concluded that MP significantly improves subsequent (surgical) performance.

Improvement in skills were perceived to encapsulate two broader areas: 1) technical and 2) non- technical skills. Technical skills were perceived to be motor skills which enabled actions, such as surgical movements and flow. Non-technical skills included skills which underpinned actions, such as communication, coping strategies and team-work.

There are marked differences in designs, research protocols, training regimens, populations and outcome measures among the studies reviewed. Despite this heterogeneity, positive effects of MP on both technical (motor function) and non-technical skills (stress and confidence) were reported. All of the reviews found some strong evidence for the positive impact of MP on Levels 2a and 2b of the Kirkpatrick framework.

It does appear therefore that surgeons can benefit from exercising and honing this skill both at novice and experienced levels of practice. We concur therefore with the findings in the comprehensive review by Sevdalis et al. [41] that appreciable evidence exists to support further investigation. However, there are a number of outstanding questions and the limitations of this review should be considered alongside its findings.

### Review limitations

We used MP as an umbrella of mental imagery, mental simulation and mental rehearsal as these key words (and others) were reported across the reviews as search terms (Table 3). However, this encompassing conception may risk skewing more highly focussed definitions and their findings [44]. Mental practice is concerned with mental processes and can also involve all the senses, but in some studies the focus was on visual mental imagery as most empirical work has addressed this sensory domain [48]. Nevertheless, evidence demonstrates that these sense modalities interact in a variety of ways [49, 50] and there is still incomplete knowledge regarding the degree of interplay between broader cognitive skills and a narrower focus on psychomotor skills.

The critical issue here is the heterogeneity of mental representations which points to practical and theoretical reasons why mental practice is challenging to study in whatever way we choose to decline it. Furthermore, a detailed understanding of what MP comprises, by its very nature, can remain an illusion. In agreement with Schuster et al. [40] we chose a broad and inclusive working definition which can be understood as an interaction between cognitive, perceptual and motor systems, but acknowledge this may be less precise than among other reviewers.

Despite an inclusive search strategy around mental practice as ‘simulation’ we did not include technology-based simulation approaches to priming performance. Excluding grey literature and non-peer reviewed articles may have overestimated the quality of the literature field in medicine. This however supports findings that the MP and MI are inadequately defined.

Poor reporting in primary studies may also have led to an unduly negative assessment of quality of the reviews. The depth of impact, i.e. along the Kirkpatrick model, was relatively limited. The preponderance of RCT designs [51] in the reviews may have restricted the analysis of more enduring examples of acceptance, learning and acquisition of skills thus explaining the inability to reach higher levels on the Kirkpatrick scale. Surprisingly however, and even when the focus of primary studies was narrower, what exactly MP comprises was insufficiently described to make the interventions easily reproducible.

A further criticism could concern our choice of an outcomes-based evaluation framework. In fact, to guide our abstraction of the different learning outcomes of MP, we used Kirkpatrick’s model of educational impacts. This model offers a pragmatic four-point description of educational outcomes which is followed by the Best Evidence in Medical Education group (BEME) [52]. In our analysis of outcomes with Kirkpatrick’s framework we do acknowledge some conceptual limitations.

A criticism of Kirkpatrick is that the use of levels concept implies the framework is about a product, a particular learning outcome of interest, rather than a process [53]. We agree the process is important because it illustrates the quality of teaching and learning, directed and self-directed, needed to achieve the outcome. However, we believe this is not subverted by the model. In an influential review of skill acquisition Wulf et al. [54] make the important distinction between learning and performance. The papers in the reviews privilege the latter by comparing MP with no mental practice on immediate or very short-term performance outcomes. These studies attempt to evince how performance is influenced by a particular training method. This may or may not have anything to do with how much was learned. In other words, performance and learning can be uncomfortably aligned. The reason is because learning is a relatively enduring transformation in a person’s capability to perform a skill. Significantly, learning also shapes how we think about how we learn and prepare for performance. These metacognitive adjustments shore up retention and resilience over time. Surprisingly, and despite comparisons with expert practices, the value adjudicated to mental practice was largely restricted to a specific and time-limited skill performance, rather than acceptance (by participants), or retention and transferability of the MP ritual by participants to other clinical skill contexts. Given the accumulated evidence of MP benefits outside of healthcare, whether and how users apply MP post training is surely a key researchable impact. Remarkably, however, only half the reviews mention its desirability.

A further and final criticism is that Kirkpatrick levels delineate a patchwork of different beneficiaries: levels 1–3 concern learners (where reviews reported positive improvements), but levels 4a concerns organisations and level 4b concerns patients. Perhaps critically, teachers are missing from the scheme altogether [55]. Despite these limitations of the model, we believe it does invite evaluators in systematic reviews to think about what happens afterwards, and longitudinally. This implies prospective evaluators should consider evidence for sustained changes in individual and collective practices, including the frequency and exposure to independent user follow up of MP and how this is supported in practice and policy in the organisation. And not simply in training curricula re-design, but for instance in the time and space allocated to MP in pre-surgical routines and briefings. The impact of sustained MP on surgical flow and its effects on patient care should also be analysed.

Evaluation researchers therefore need to think of the longer term and not simply the immediate performance. In particular, they need to identify different methods of data collection and a variety of sources within the umbrella levels which could throw light on different impacts.

### Implications

At first glance it appears that MP is still largely in thrall to the taxonomy of psycho-motor skills and step-wise mental imagery. The obverse side however is that our human capacity for reflection, self-awareness, and meta representations, the ability to have also a concept of the mental representations of others, invites a broader set of propositions about MP and learning in educational contexts [4, 56–59]. Mental practice (and mental imagery) allow us to visualise, remember, plan for the future, navigate and make decisions. Quintessentially, what emerges from a number of reviews is that MP harbours a logic of ‘expertise’ [36, 38, 39]. It postulates, for instance, that through guided mimicry of expert routines less experienced surgeons can be inducted to practices that help bridge the gaps, thereby shortening their learning trajectories. Consequently, this lends substance to the idea that we should inquire into the pedagogic, learning and cultural conditions that might render the practice of MP more likely over time. Implicit in this view is that MP itself is a key transferable skill. That is, it can be practised, learned and improved upon. From this standpoint we believe that MP is still under theorised and little acknowledged.

This brings us to the following related point. MP assumes that practitioners already have ‘imaginative’ resources that are critical to quality and safety but that these resources need to be supported organisationally – for example by being instantiated in pre-surgical routines. This requires organisational spaces, leadership and involves a kind of interpersonal expertise. Interestingly, recent research suggests that teams can share their practical envisioning derived through MP with others to improve their collective performance [15, 60, 61]. In fact, MP posits that this sharing of an individual’s ‘mental rehearsals’ – at expert but even novice levels - can lead to better performance and enhanced safety in the team simply by dint of its communicative and rhetorical force [15, 60, 62, 63]. Organisational researchers have referred to these interactions as ‘zones of collaborative attention.’ [64] Mental practice, in other words, can also be an ‘information sharing’ practice which is proximal to the task and imbricated with important and familiar notions in surgery of ‘shared mental models’, ‘anticipatory thinking’ and ‘situational awareness.’ There is a difference of emphasis here with mainstream educational notions of ‘reflective practice’: MP privileges cultivating ‘foresight’ over retrospective review or ‘reflection on action’. [65] Future research in learning and improvement science needs to address these dimensions of MP and their contributions to how safety is achieved.

In consideration of these factors, in designing procedures for surgical training, surgical team building and surgical safety, MP has potential to improve performance and its application is highly economical, sustainable and feasible with current resources.

## Conclusions

Although the literature has burnished attempts to apply MP to surgical education, there is a need for more thoughtfulness about evaluation methods. These should explore not only the immediate effects on skill demonstration, but broader notions of acquisition and, importantly, uptake of MP among users over time to enhance their performance.

Beyond the individual, training interventions should aspire to instigate similar change across the knowledge, skills, attitudes and behaviours of teams and organisations. Clarity on these micro and meso-level objectives we believe are essential in the design and delivery of effective educational interventions and also their evaluation.

There are strong implications too for the social function of mental rehearsal in teams and its contribution to safety. MP can potentially shape the way a surgical team acts as a ‘collectivity’ which is critical to how safety is accomplished [66, 67]. Determining the most influential shifts in practice, at the individual, team and organisation levels will require more validation in future studies using more sophisticated and longitudinal research designs.

## Supplementary information

**Additional file 1: Appendix 1.** Supplementary Data

**Additional file 2.** Search Strategies

## Data Availability

All data generated or analysed during this study are included in this published article [and its supplementary information files].

## Abbreviations

FLS: Fundamentals of Laparoscopic Surgery Simulation
HRV: Heart Rate Variability
LC: Laparoscopic cholecystectomy
MHPTS: Mayo High Performance Teamwork Scale
OSATS: Objective Structured Assessment of Technical Skills
PSCE: Objective structured clinical examination
OTAS: Observational Teamwork Assessment for Surgery
ISAT: The Imperial Stress Assessment Tool
GSOP: The Global Scale of Operative Performance
SRC: Surgical Coping Scale
STAI: State-Trait Anxiety Inventory
VH: Vaginal Hysterectomy
VR: Virtual Reality
MIT: Visual Imagery Test
OR: Operating Room
