# Supplementary material for: Critical analysis of evidence about the impacts on surgical teams of ‘mental practice’ in systematic reviews: a systematic rapid evidence assessment (SREA)
Source: BMC Med Educ. 2020 Jul 14;20:221. doi: 10.1186/s12909-020-02131-3 (PMC7362567; doi:10.1186/s12909-020-02131-3)
Supplement: Supplementary file 1 — Additional file 1: Appendix 1. Supplementary Data [file 12909_2020_2131_MOESM1_ESM.docx]

Appendix 1: Supplementary Data

**Narrative Synthesis of the included Mental Simulation Reviews**

*SYSTEMATIC REVIEW 1*

*AMSTAR 2 –* Quality Assessment: ***‘MODERATE’***

*Davison et al. (2017) Mental Training in surgical education: a systematic review*

*Key Words*: ‘mental training’; ’mental rehearsal’ ‘cognitive training’; brain training’

*Aims*

These authors report on a review which aimed to investigate the role of Mental Training (MT) in surgical education. They define MT as “the act of mentally rehearsing a sequence of motions in the absence of overt physical movement”

*Rationale*

The authors’ rationale for MT is that alternative training environments including simulation techniques are needed to meet new challenges. These include changing surgical fields such as ‘minimally invasive surgery’, work time regulations, and patient safety concerns. New learning environments however present new kinds of problems including cost, on going technological development (e.g. VR) or heterogeneity in assessment tools (e.g. box trainers) hence MT offers a path s to strengthen learning opportunities.

*Theoretical Stance*

The authors’ theoretical anchors derives from neuroscience, especially the ‘neurophysiological correlate ‘of MT in the acquisition of motor skills, attentional stability and self-confidence through the ‘activation of new neural pathways’.

*Search strategy*

The authors searched EMBASE and MEDLINE for studies in English published between 2004-2015 using “mental training”, “cognitive training”, “mental rehearsal” and “brain training” combined with ‘surgery’. Inclusion criteria was any form of Mental Training in surgical education of medical students to expert surgeons RCT and non RCT studies.

*Screening and selection*

Following the screening of abstracts and full articles 14 studies were selected( 11 RCTs and longitudinal trials

*Data Analysis*

MT activities ranged from facilitated group tasks to one-to-one with MT educators as well as use of MT prompt tools including hand-out about MT and CD rom to combinations with relaxation exercises and hypnosis. The major focus was on surgical performance. Only one study looked at outcomes in laparoscopy, but with medical students. Tasks following MT ranged from box trainer laparoscopy to VR to live surgery on anaesthetised animals. Most studies focussed on skill acquisition amongst lesser qualified medical personnel.

Nine of the studies applied ‘mental rehearsal “ as the commonest form of MT”. This was done ‘in group settings (with collated results) and 3 were one-to-one with an MT educator. Seven of nine found significant benefit and 2 of three one to one interventions found significant benefit, and one no difference.

Of the studies included 11 where RCTs and 2 were longitudinal studies. All eleven included studies used a control group. Tasks included VRS or box trainers or both and 2 studies included live surgery. Seven studies reported improvements in the MT group. Two studies reported improvement in the MT group using surgical performance checklists (OSATS and Modified Task Specific Checklist) to score MT and non-MT groups. Three other studies also reported over all surgical improvements in the MT groups using a pre-post test. One other study reported improvement in ATLS performance and one in peg transfer times. Four studies reported no improvement in the MT group with one of these studies reporting poorer performance

*Outcomes*

In regard to the outcomes Davison et al. report 7 studies reported improvements in technical performance or overall surgical performance. Three studies reported improvements in self-confidence, knowledge of procedure and reduced anxiety. Two studies reported improvements in participants’ mental imagery using the MiQ tool. One study correlated the improvement in MiQ to surgical performance

The majority of studies were focussed on changes in surgical performance and 9 recruited surgeons, of which 3 experiences and 6 studies novice surgeons. Authors report that only three of the 11 RCTS met quality standards using the CONSORT tool (>16). Two of which reported benefits of MT. Authors report that the 2 longitudinal studies also scored poorly using a published quality assessment tool.

*MT consensus*

The programmes vary greatly in terms of content and duration. Four studies provided no information on the duration of MT sessions but others ranged from one study of 5 minutes and 4 studies of less that 30 minutes of MT. Six studies involved >30 minutes of engagement. Whether shorter or longer exposure impacts performance more is unanswered in the systematic review. Papers examining non-technical aspects in outcomes not considered in this review – either excluded or not found. Only 5 studies provided sufficient detail for replication – e.g. MT protocols were unclear, follow up unclear, timings incomparable.

Heterogeneity of outcome measures made studies incomparable. Five studies based their assessments on peg transfer times

Programme quality is a key evaluation component missing in the studies analysed.

*Conclusions*

Based on their findings the authors offer the following conclusions : “the results could suggest an MT to be a beneficial component of surgical education”. Secondly MT is more beneficial for strengthening the cognitive component (e.g. task specific checklist compliance) of practice, although the authors ignores the social cognitive realm of practice. Thirdly, the benefit of MT is greater in more expert surgeons and this replicates evidence from MT in elite sports. Lastly, the authors advocate the inclusion of MT in the surgery curriculum at intermediate/advanced levls for procedure specific or full procedural training with web lab simulation and real practice.

None of the studies explore participant feedback beyond Kirkpatrick 1-2. This is curious as when is a surgeon ready to operate on a person is a vital question that each participant must have a voice in deciding (along with their supervisor). Primary studies did not explore this dimension of expertise. Finally Davison’s definition of ‘cognitive training’

Cognitive behaviour change – re-structuring of team cognition through MT surprising that Davisson’s focus on cognitive training is confined to surgical knowledge ignores related cognates in particular the social behavioural realm. The role of MT in collective safety is unexplored.

*NON SYSTEMATIC* *REVIEW 2*

Hall, JC. (2002) Imagery practice and the development of surgical skills

*Key Words*: ‘surgery’; surgical education; surgical training; cognitive psychology*; motor skills

* ‘mental rehearsal’ used in text as cognate of ‘mental imagery’

*Aims*

Hall writes that the purpose of his review is “to explore the potential role of imagery practice during the acquisition of surgical skills” and to “raise awareness” of the role of MI in the “training” and “maturation” of surgeons. Although he refers specifically to the acquisition of complex motor skills, Hall is interested in broader transfer of learning and MP as a form of self regulation.

He defines ‘imagery practice’ as the “symbolic rehearsal of a physical action in the absence of any gross muscular movement”. But adds learning theory in particular clarity of goals, exploration of how to perform the task, and then practice so that it becomes routine.

*Rationale*

MI shares neural networks with major cognitive functions such as language, memory, and movement depending on the nature of the task. “Hence, there is no unique mental imagery neural network” There is a “high degree of interaction between mental imagery and other cognitive functions” The human brain is able to create “ a highly edited subjective version of the world” as the substrate for MI

*Theoretical Stance*

Neuroscience – and functional reorganization within the primary motor cortex, and Cognitive psychology self regulation and reflection. (individual!)

*Search Strategy*

Searched MEDLINE between 1980-2001

*Screening and selection*

None – this is a narrative review

*Data Analysis*

No critical assessment of primary studies

*Outcomes*

For inexperienced trainees, MI is a mechanism to ‘cogitate’ what is involved in the insertion of ports; more experienced trainees might go over the steps of the whole operation; more experienced trainees to go over the steps of an op they have observed but not done previously. Experienced surgeons could use MI to ‘rehearse’ variations to answer what if scenarios. In this safety perspective – mindfulness perspective – Hall refers to ‘risk management’

*MT consensus*

Hall’s cyclical six stage technique of MI reflects well described education practice to prepare for any learning event:’ task definition’; ‘prior learning’; ‘mental rehearsal’; ‘reflection’, ‘problem solving’; ‘reality check’ (or *feedback*)

*Conclusion*

Interestingly, Hall associates MI with reflective practices and self-debriefing in the development of skill development. His reference to ‘risk management’ is the most interesting observation of experienced surgeons.

*SYSTEMATIC REVIEW 3*

Rao et al. (2015) Systematic review and meta-analysis of the role of metal training in the acquisition of technical skills in surgery.

AMSTAR 2 – Quality Assessment: **‘HIGH’**

*Key Words*: ‘mental training’; ‘mental imagery’; ‘technical skills’

(author lists synonyms as: ‘mental rehearsal’, ‘mental practice’ – ‘visual imagery’, ‘motor training’, ‘auditory imagery;, ‘olfactory imagery’ and ‘motor imagery’)

*Aims*

These authors report on a systematic review and meta analysis which aimed to evaluate the effect of MT on the learning of surgical technical skills. They define ‘mental training’ as “rehearsal of mental imagery without physically performing the task” (to ‘view and feel the task without doing it.’)

*Rationale*

To face challenges of training – reduced hours, changing skills – new educational spaces are needed and ‘mental training provides a potential alternative for surgical units to acquire technical skills’. Rao, et al. also draw on expertise research and the experienced surgeon’s “mind’s eye”. A part from technical skills, the authors also refer to the role of MT in ‘tutoring’ trainees.

*Theoretical Stance*

Neurophysiology (rehabilitative medicine & sport, music) Roa et al. make reference to ‘dual coding’ citing Hayter.

*Search Strategy*

The authors searched EMBASE, MEDLINE, Web of Science, Clinicaltrials.gov.uk, SIGN guidelines, NICE guidelines, and Cochrane Review register between 2004-2014. The authors used a combination of ‘mental practice’, ‘mental imagery’ ‘visual imagery’, ‘motor training’, ‘auditory imagery’; ‘olfactory imagery’ and ‘motor imagery mental training’ and identified further studies through cross-reference of reviewed papers. Inclusion criteria were: RCT assessing the role of MP on surgical training; participants with ‘medical background’ (surgeons, surgical trainees, medical students, trainers); articles on any aspect of mental imagery (see key words); articles that measured any outcome; articles on any MT approach (script memorization, psychotherapy; hypnotherapy etc)

Excluded articles were ones which were non experimental

*Screening and Selection*

Following the screening of abstracts and full articles 9 RCT studies were selected.

*Data Analysis*

Total of 474 participants of which 189 received MT. Six of the 9 RCTs included recruited medical students. Three RCTs had surgical trainees. RCTS were scored using the JAHAD scale. A variety of assessment tools were reported and all of the studies used direct observation measures. Of the 3 surgical studies using trainees, 2 used the OSATS to score technical performance on a virtual laparoscopic cholecystectomy simulator and one on a pelvic box with pig liver and gall bladder. The third used a psychometric objective test on group mental rotations plus MiQ and Stress assessment measures against performance on a laparoscopic cholecystectomy simulator. The 6 studies with medical students used 6 different measurement tools in tasks relating to: (1) cut out tasks on box trainers (measuring time, precision), (2-3) incision and suturing of live rabbits (a binary checklist, a standard test of mechanical-spatial abilities, plus an attitude scale and, in a second study a Z-score – or number SD from the mean), (4) cutting a circle on a laparoscopic trainer and VRS assessment (accuracy, precision);(5) a simulated knot tying task (time, trajectory, scale); and (6) a simulated cricothyroidotomy procedure (Objective Structured Clinical Exam)

*Outcomes*

Three studies with surgical trainees reported favorable outcomes. Four studies with medical students showed no significant effect while two further studies with medical students showed positive effects.

*Conclusions*

Based on their findings the authors offer the following conclusions: Firstly, the significant heterogeneity of study methods and MT from/content made a meta analysis impossible. Secondly, the studies which showed no effect had shorter times of MT and unsupervised practice tasks that participants were required to conduct in their own time; furthermore, these were studies with undergraduate medical students. Repetitive sessions 30-90 minutes in addition to physical practice with surgical trainees using cue scripts were the most effective. Thirdly, the mode of MT was underspecified in most studies and whether MT was done individually or collectively was unclear. Similarly, the order of MT v physical practice was not described in most studies. Finally, Roa et al. make the important point that only ‘short-term’ effects of MT were analysed. The authors say that organizational effects of learning and practice are ignored in the primary studies and that their generalizability is limited due to small sample sizes.

*SYSTEMATIC REVIEW 4*

Anton, N.E et al (2017) Application of mental skills Training in surgery: A Review of its effectiveness and proposed next steps

AMSTAR- 2 Quality Assessment: ***Low*.**

*Key Words*:* Surgeons, Surgery, Mental Skills, Mental skills training, Mental training, Performance enhancement, Stress, Stress management Training

* For searches: *mental readiness, mental competency, mental skill, cognition, mental practice, imagery, mental imagery, mental rehearsal, sport or performance psychology, stress management training, stress coping, mental training, performance enhancement, surgery, surgical performance, medicine, and medical competency*

*Aims*

The authors undertook the review to “identify how mental skills training has been applied to

surgery” to compare it to other domains (outside medicine) and to assess the evidence for its effectiveness in enhancing surgical performance and reducing stress. The authors also aim to identify the “potential benefits’ of successful mental skills training from other domains.

The authors define ‘mental skills’ training as a ‘psychological strategy’ for the ‘teaching of performance enhancement and stress management’

*Rationale*

The authors report their previous survey that 40% intraoperative complications were related to surgeon stress. They contend that ‘structured mental training’ may benefit surgeons as an additional method to strengthen mental readiness, confidence, focus and constructive evaluation, as new surgical techniques evolve. The authors also quote studies on MT curricula in sport , music and the military which described improvements beyond single skill interventions “ as they aid performers’ flexibility to manage a variety of situations”

*Theoretical Stance*

Psychology (individual Zone of Optimal Functioning – IZOF )and adult learning theory on ‘curricula; design (not explicitly elaborated upon) to provide the intensity of exposure to MT techniques to improve surgical performance over time.

*Search Strategy*

The authors searched Medline, PubMed, PsycINFO, and ClinicalKey data bases between 1996-2016.

*Screening and Selection*

490 abstracts were reviewed by two authors. Articles were included if the primary outcomes of mental skill intervention was surgical performance in a simulated environment or in the OR ‘independent’ of participant type (e.g. medical students, surgical trainees, experienced surgeons). Articles were excluded if the primary outcome was not surgical performance, or if the intervention was related to the impact of non-mental skills on surgical performance or stress (e.g. music in the OR). Publications from sport were included to identify different applications of MT possibly relevant o surgery. 28 studies were identified. Of the included studies 18 were RCTs, 1 a prospective quasi experimental study, 4 reviews, 2 position papers and one commentary.

*Data Analysis*

Twelve of the RCT reported positive effects on their combined knowledge, confidence , kinaesthetic experience (1 study) compared to no training, or improved skill acquisition and performance compared to a control group (8 studies): Other positive outcomes included teamwork (2 studies) Error reduction (1 study). Two studies with surgical residents showed no significant differences in surgical performance compared to a control group (2 studies), although in one of these participants rated imagery practice as ‘highly effective’ and the other improved confidence.

2 RCTS with medical students reported equal benefit of physical practice followed by imagery training on suturing exercises and one of these reported effectiveness in ‘transfer’ of surgical skills.

One RCT reported no statistically significant trend towards enhanced technical performance after stress management training while the second saw no technical improvement with medical students on time and accuracy of laparoscopic tasks.

The 4 reviews and position papers supported the use of mental practice for explicit learning of surgical skills (2) as a supplemental training tool (1) as a low cost and flexible curricula addition (2)

*Authors Conclusions*

Based on their findings the authors conclude that in non-medical domains ‘mental skills or trainable psychological techniques’ have been show to enhance performance and attenuate declines in response to stress. They make four conclusions. Firstly, mental skills enhance surgical performance and skill acquisition, increase knowledge, confidence, team-bases skills and reduce stress. Secondly, most definitions of mental training concern ‘mental imagery’, However, relaxation strategies, performance routines, goal setting and management skills ‘which have been highly effective in other disciplines” may be useful too.

*SYSTEMATIC REVIEW 5*

Schuster et al. (2011). Best Practice for motor imagery: a systematic literature review on motor imagery training elements in five different disciplines

AMSTRAR -2- Quality Assessment: **HIGH QUALITY**

*Key Words:*

*Mental imagery; mental practice; mental rehearsal; mental movements; eidetic imagery; visual imagery; guided imagery; motor imagery; mental training*

*Aims*

The authors state that the aim of the review was to identify the characteristics of ‘mental imagery training sessions’ (MITS) ‘with *positive* results’ and compare these for different disciplines, session types, task focus, age and gender and MI modification during intervention as well as to identify ‘fundamental intervention designs’. It was not the aim of the authors to evaluate the effectiveness of MI but to ask “how should MI be done?’

*Rationale*

Authors cite Evidence in sports that MP can accelerate learning and improve motor skills. Schuster et al define ‘mental practice’ as an ‘umbrella term that includes various mental training interventions’

Theoretical Stance

Functional neuroscience literature - Realising the need for a theory and research-based model of imagery to help guide practitioners’ use of imagery, the authors refer to Holmes and Collins (2001) PETTLEP model*. This model is based on theory and research findings from sport psychology, cognitive psychology and neuroscience, and aims to provide practitioners with a set of practical guidelines to aid their imagery use: the reviewers used the categories – Physical. Environment, Task, Timing, Learning, Emotion, Perspective – to extract MITS from the primary studies

*(By convention, nowadays, a theoretical representation with rather specific or restricted scope tends to be called a model, whereas a theoretical representation with broader scope tends to be called a theory - HS)*

*Search Strategy*

The authors searched PubMed, Google, Google Scholar, cross-checking with MeSH terms between 1996-2016. Using SCOPUS they searched a total of 24 databases.

*Screening and Selection*

The authors included quantitative intervention studies in multiple disciplines focused on motor skill, performance or strength improvement. They excluded VR, hypnosis or MP not related to movements. After screening processes were employed, 133 articles were located, of which 37 in medicine, 9 in Education, 5 in music, 82 psychology and 10 in sport . The studies originated in Europe, Australia/NZ, the Americas, Asia and the Middle East. (91 RCTs, 22 Controlled trials, !5 case series, 13 case studies)

The studies in medicine were all to do with patients and MP as part of rehabilitation. Instead, the 9 studies classified under education were all in medicine and nursing, of which two only for graduates (Surgeons). See Table 3.

*Data Analysis*

The authors used published rating scales (e.g. from physiotherapy) to rate the quality of studies. The PETTLEP model to extract MITS from primary studies. Owing to lack of reporting, the ordering of MI/PP could not be identified in 90% of all MI interventions (in Education –medicine ! – MI performed predominantly before PP. MI interventions with positive results had almost twice the duration of MI interventions with no change or negative results. The authors studies described activities which were mainly motor focused (94), cognitive focused (29) and strength focussed (6). Cognitive focussed activities had shorter duration times. Of the 37 positive MI sessions reported in groups (v individual MI sessions) they were mainly directed and embedded in PP and included MI practice before and after PP. 129 interventions reported positive results and only 12 MI studies reported no change or negative results. The analysed studies primarily investigated the short-term effect of MI with a simple pre-/post-test design. The longest time period evaluated was a 6-month follow up in an RCT on pain management for patients (Moseley et al ) Over all, data reporting in the selected studies was ‘low’. The authors highlight, for example, that ‘imagery perspective’ was least reported.

Authors’ Conclusions

The MI interventions were heterogeneous and there were large SD in temporal parameters. The author state that before applying an MI interventions, “it is essential to evaluate the MI ability of the participants to determine whether they are able to perform MI” .However, the heterogeneity of MI assessments used prevented direct comparisons.

The authors conclude that “reports on MI interventions did not use consistent terminology, and often lacked details on MITS elements and temporal parameters” and that “Further reviews should consider the influence of MI on psychological factors, such as goal setting, self efficacy, motivation and mood’

*SYSTEMATIC REVIEW 6*

Cock et al. *What surgeons can learn from athletes: mental practice in sports and surgery*

AMSTAR -2- Quality Assessment: **MODERATE**

*Key Words:*

*Mental practice, imagery, surgeons, experts, surgical skills (mental rehearsal, motor rehearsal)*

and

*Competencies:* *‘practice-based learning’ learning and improvement’ ‘medical knowledge’ and ‘patient care’*

*Aims*

The authors state that the aims of their review are to describe the literature on ‘mental practice’ in sport psychology and surgery and to explore how the principles of MP can be applied to the improvement of, not only technical skills, but also surgical *performance* in novice and expert surgeons.

The authors define mental practice as ‘the conscious action of systematically and repeatedly imagining objects and movements without physically seeing or performing them with the intention of improving performance’

*Rationale*

‘Sport and surgery both deal with execution of orchestrated fine and gross motor skills in a time sensitive, high stakes environment’ hence surgical education can take example in sport of MP to expand the cognitive tools available to learn and enhance performance. There is also very little data available on MP to enhance established surgical skills

*Theoretical Stance*

Cognitive psychology (see S. Kosslyn) and expertise research (see Ericcson)

*Search Strategy*

The authors searched PubMed, Medline, PsycINFO and Embase for English language papers in sports, psychology and surgery using the following words individually or in combination: ‘mental practice’; ‘mental rehearsal’; ‘visual imagery’; ‘surgery’; ‘sports’. The authors searched reference lists and expanded search to medicine and music when related to MP.

*Screening and Selection*

40 articles ‘relevant to mental practice’ in either sports or surgery were retrieved. The only articles listed in the review concern medicine and they consist of 10 RCTS. No information is provided on how papers were included or excluded.

*Data Analysis*

The authors structure the review firstly on MP in Sport, then MP in medicine with a further subdivision between MP impacts on novice and expert surgeons.

*Outcomes*

The authors report findings from sport - Feltz and Landers – which indicate that MP is more advantageous when used by elite athletes with a combination of physical and mental practice. But also that MP could be deleterious in novice athletes who do not have the basics (Suinn 1997)

In medicine 8/10 studies showed positive effects of MP. With temporal differences of between 3 and 90 minutes. Outcomes included residents perception of improved preparation, improved observational teamwork, coping skills, quality of novice surgical performance, better visuo-spatial ability, improvement when compared to task specific checklists in laparoscopic simulations, improved MI and transfer to practice. Tasks ranged from suturing skills (2)

laparoscopy (4), vaginal hysterectomy (1), simulated carotid endarterectomy (1) cystoscopy (1) and cricothyrotomy (1). One study (Bathalon et al.) with 44 medical students at 12 months found better OSCE score on cricothyrotomy in the MP group (MP +kinesiology) than control group (ATLS)

Among expert studies, two were non RCT interview studies that showed that physicians often used mental images – radiology etc – to recall patient cases (1 study) and that surgeons thought ‘mental readiness’ was important to be able to envision good outcomes, positive imagery and positive thinking.(1) MP was also reported in an RCT to reduce stress and improve contingency planning in preoperative briefs (Arora) and ‘proactive coping’ (Wetzel) and NTS scores.

The authors state that many trials used different kinds of mental practice with considerable variations in duration, timing, frequency, of MP sessions. The content and delivery also ranged from validated MP scripts to facilitated rehearsal to relaxation sessions. Most of the studies have small sample sizes and may not have power to produce reliable results.

*Authors’ Conclusions*

‘Our review suggests that MP may be used to optimise surgical performance for surgical trainees and expert surgeons alike.’ The authors state it should not substitute PP but that it may offer important psychological advantage as in elite sports. They also suggest that RCTs may not provide sufficient analytical grit to answer the hypothesis regarding the benefits of MP in surgical practice – rather, they suggest that future research should include exploratory naturalistic and ethnographic studies to answer the question why experienced surgeons use MP to enhance or maintain performance.

In comparing sport to surgery the authors ask how the athletes’ MP to remain in control of their environment might transfer to surgery, where the environment is perhaps less predictable (?!).

*SYSTEMATIC REVIEW 7*

Marcus et al (2013) Practice makes Perfect? The role of Simulation-based deliberate practice and script-based mental rehearsal in the acquisition and maintenance of operative neurological skills.

AMSTAR -2- Quality Assessment:

Review excluded after consensus discussion.

Key Words

*Deliberate practice, mental rehearsal, neurosurgery, scripts, simulation-based medical education*

*Aims*

The authors state that the aims of the study are to “discuss the theoretical arguments for simulation-based learning and script-based mental rehearsal in neurosurgery and the evidence for their efficacy in allied surgical fields”

The authors, citing Sanders, define ‘script-based rehearsal’ as ‘the cognitive rehearsal of a task in the absence of overt physical movement in which trainee surgeons are given scripts specially formulated by experts….’ ) i.e. ‘they are given a gold standard’

*Rationale*

Surgical education is undergoing overwhelming change, in part as a result of changes in economic, political, social, cultural, and technological climates in which it operates. Shortened training, reductions in the working week, economic restraints, and increasing emphasis on patient safety have required surgical educators to radically rethink how surgical education is delivered. The authors make the link with Ericsson’s ideas of expertise development – including the enhanced capacity of experts to envision scenarios.

*Theoretical Stance*

Cognitive psychology, ‘schema theory’, ‘staged motor learning’ – deliberate practice (increase in skills for all levels of expertise) - inconsistent referencing.

*Search Strategies*

None

*Data Analysis*

Reports only positive studies from sport and surgery.

*Outcomes*

Positive only – transfer of learning

Authors’ Conclusions

The evidence base in both surgical and non-surgical disciplines suggests that mental practice may be a cost-effective and safe way of training novice surgeons.

*SYSTEMATIC REVIEW 8*

Sevdalis et al. (2014) Mental Imagery and Mental Practice applications in Surgery: State of the Art and Future Directions

AMSTAR 2 - Quality Assessment: **High Quality**

Key Words: *mental practice; mental imagery, mental rehearsal, surgery, surgical education, operating room, operating theatre*

*Aims*

The authors state that the aim of the review is to provide “a state-of-the-art review” of the application of mental imagery and mental practice (or motor imagery) within surgical contexts and to summarise the main theories of “mental practice effects”

The authors define ‘mental imagery’ as a form of cognitive simulation, and ‘mental practice’ as the covert rehearsal of an action in one’s imagination without executing the actual movements involved.

*Rationale*

Research on mental simulation processes such as mental imagery (or mental practice) provides an empirical window on movement planning and through mental practice facilitates skill learning and skilled performance in sport. This has implications for surgical training. The impracticality and pedagogical challenges of the apprenticeship model which is learner centred, working time pressures and cost efficiency make alternatives to traditional surgical training attractive. Hence the interest in MP as a tool to enhance simulation-based training.

*Theoretical Stance*

The authors provide an extensive review of the theories of mental practice, including the neuromuscular model (Jacobsen 1932) the cognitive or symbolic approach (Denis 1985) and the bio-informational theory (Lang 1979)

*Search Strategies*

The authors used 2 data bases; They searched Medline and PsycInfo from earliest entries to 2012. Search terms included “mental practice/rehearsal/imagery/training (including synonyms) and hits were filtered using the terms “surgery”, “operating theatre/room and ‘operative’ using synonyms. The reference lists of retrieved articles were also hand-searched for relevant references. Retrieved articles were included in the synthesis if (a) reported original research (b) reported research on surgical tasks or performance. Thirteen articles were included: 10 RCTs 2 qualitative studies and one pre post intervention. Seven of the papers included surgical trainees or surgeons and 6 involved medical students.

*Data Analysis*

According to the review only 2 studies utilised an MP immediately prior to a surgical task with surgical novices that had some evidence of validity (Arora 2011 a b) comparing technical performance and stress levels (subjective and objective) with controls. The authors showed improvement in surgical imagery using a manipulation check validated MIQ tool prior to examining the impact on the task. None of the other studies used a validated MP tool and so compliance with imagery instructions was impossible to determine. Four of these studies reported benefits in ‘enhancement of performance’ the findings could not be used as evidence for the validity of MP. The authors point to mixed techniques as confounding factors – e.g. mixing kinesiology with MP -; lack of between subject comparisons and other experimental groups (Immenroth 2007) and Wetzel et al (20110 which delivered MP within a larger stress coping strategies training programme where the results showed no improvement in technical performance, but improved stress coping strategies.. “This renders any direct attribution of MP effects problematic’ Three other studies with medical students reported no effects of MP on performance but had no manipulation checks and small sample sizes.

There was also wide variability in timing of MP activities and task. Only three studies used MP immediately prior to the task and one within a 24-48 hour period, Sapiens and Rogers recommends a 24h max period. In all the other studies reporting no impacts the timing as either unclear of exceeded 24h. significantly weakening the potential effects of MP

*Regarding NTS*

The timing of the only study looking at teamwork (Wetzel 2011) is unclear. Nor did Wetzel use an MP manipulation check tool. They used a well-validate team performance (OTAS – Observational Teamwork Assessment for Surgery’ tool) but the study revealed no improvement. The other methodological flaws could explain the lack of effect, including the programme of stress management.

Stress was also examined in Arora et al and Wetzel with mixed results using a stress test ISAT; Arora found improvement while Wetzel di not

*Regarding technical performance*

Technical performance, namely psychomotor execution of the surgical task ws measured using OSATS in 3 studies 9Arora, Wetzel, Immeroth). Two other studies used validated global rating scales. Surgical performance was also capture by the virtual reality simulator including time to complete task, instrument trajectory length in endoscopy, Only one study with medical students did not use a validated measure!

*Outcomes*

Three studies reported found that MI of both junior and senior surgeons improved significantly following MP training.

Two qualitative assessments of mental preparedness prior to a procedure found that surgeons considered mental preparedness to be at least as important as their technical readiness (McDonald and Orlick 1994 McDonald 1995)

Five studies found no technical improvements on a range of technical measures while 5 found improvement .

*Conclusions*

The authors take an individual skills approach to MI; nevertheless the authors call for performance assessment that goes beyond purely technical skills to encompass non-technical aspects of performance, including leadership communication and stress management; available evidence base is small but has grown rapidly in recent years. Most studies are hampered by methodological flaws. The majority of studies had no MP manipulation checks. Time lag was problematic because unclear plus the use of non-validated instruments. IN the robust studies MP enhanced the technical performance of typically junior surgeons. The qualitative studies with senior surgeons consider MP as an important determinant of surgical excellence and call for its inclusion in surgical training. A final methodological point regards the lack of a gold standard regarding ‘mental imagery’ the risk that the personalised attention and in-depth reflection towards individual in MP creates a Hawthorne effect. The authors refer to a sport ‘mental imagery index (MII)
