# Supplementary material for: Critical analysis of evidence about the impacts on surgical teams of ‘mental practice’ in systematic reviews: a systematic rapid evidence assessment (SREA)
Source: BMC Med Educ. 2020 Jul 14;20:221. doi: 10.1186/s12909-020-02131-3 (PMC7362567; doi:10.1186/s12909-020-02131-3)
Supplement: Supplementary file 2 — Additional file 2. Search Strategies [file 12909_2020_2131_MOESM2_ESM.docx]

**Search Strategies**

| **Data Base** | **Key word Combinations** | **Results** |
| --- | --- | --- |
| Medline (PubMed) | ((mental practice OR mental rehearsal) AND surgery).ti.ab | 1652 |
|  | ((mental simulation OR mental rehearsal) AND surgery).ti.ab | 179 |
|  | ((mental imagery OR mental practice) AND surgery).ti.ab | 1,700 |
|  | ((mental imagery OR mental simulation) AND surgery) ti.ab | 233 |
|  | ((mental imagery AND surgery).ti.ab | 85 |
|  | ((mental imagery AND surgery) AND “non-technical skills” | 2 |
|  | ((mental practice AND surgery) AND teamwork).ti.ab | 0 |
|  | ((mental practice AND surgery) and “surgical team”.ti.ab | 4 |
| CINAHL | (mental practice OR mental rehearsal) AND (surgery OR surgical team) | 1364 |
| Cochrane database | “mental practice” | 171 |
| EMBASE | (mental practice and surgery).mp | 25 |
|  | Mental practice OR mental rehearsal AND surgery | 40 |
| PsycINFO | (mental practice OR mental imagery) AND surgery).mp | 11 |
|  | (Mental imagery OR mental rehearsal) AND surgery).mp | 9 |
|  | (mental practice AND teamwork).mp | 2 |
|  | (mental simulation AND surgery).mp | 0 |
| ERIC | “mental practice” | 212 |
| Web of Science | “mental practice” AND (surgery OR surgical teams) | 1302 |
|  | “Mental practice” AND surgery | 149 |
